# Supplementary material for: Immunome perturbation is present in patients with juvenile idiopathic arthritis who are in remission and will relapse upon anti-TNFα withdrawal
Source: Ann Rheum Dis. 2019 Sep 20;78(12):1712–21. doi: 10.1136/annrheumdis-2019-216059 (PMC6900250; doi:10.1136/annrheumdis-2019-216059)
Supplement: Supplementary data [file annrheumdis-2019-216059supp002.pdf]

Supplementary Table S1

No.	Individuals	JIA Subtype	Gender	Medication History	CyToF	Nanostring
1	**Relapse 1011 (T_o)**	Poly RF -	F	etanercept, no MTX	✓	
2	**Relapse 1044 (T_o)**	Poly RF -	F	etanercept, no MTX	✓	
3	**Relapse 2020 (T_o)**	Poly RF -	F	adalimumab, no MTX	✓	
4	**Relapse 2042 (T_o)**	Poly RF -	F	etanercept, no MTX	✓	
5	**Relapse 4090 (T_o)**	Poly RF -	F	etanercept, no MTX	✓	
6	**Relapse 5031 (T_o)**	Poly RF -	F	adalimumab, yes MTX	✓	
7	**Relapse 6129 (T_o)**	Poly RF -	F	adalimumab, no MTX	✓	
8	**Relapse 7063 (T_o)**	Poly RF -	F	etanercept, yes MTX	✓	
9	**Relapse 8120 (T_o)**	Poly RF -	F	etanercept, no MTX	✓	
10	**Relapse 5137 (T_o)**	Poly RF -	M	infliximab, yes MTX		✓
11	**Relapse 10107 (T_o)**	Poly RF -	F	etanercept, no MTX		✓
12	**Relapse 1006 (T_o)**	Poly RF -	F	etanercept, no MTX		✓
1	**Relapse 1001 (T_{end})**	Poly RF -	F	etanercept, no MTX	✓	
2	**Relapse 1003 (T_{end})**	Poly RF -	F	etanercept, yes MTX	✓	
3	**Relapse 1006 (T_{end})**	Poly RF -	F	etanercept, no MTX	✓	
4	**Relapse 1011 (T_{end})**	Poly RF -	F	etanercept, no MTX	✓	
5	**Relapse 1044 (T_{end})**	Poly RF -	F	etanercept, no MTX	✓	
6	**Relapse 3066 (T_{end})**	Poly RF -	F	etanercept, no MTX	✓	
7	**Relapse 6049 (T_{end})**	Poly RF -	F	etanercept, no MTX	✓	✓
8	**Relapse 7063 (T_{end})**	Poly RF -	F	etanercept, yes MTX	✓	
9	**Relapse 10107 (T_{end})**	Poly RF -	F	etanercept, no MTX	✓	
10	**Relapse 3062 (T_{end})**	Poly RF -	M	infliximab, yes MTX		✓
11	**Relapse 6048 (T_{end})**	Poly RF -	F	etanercept, yes MTX		✓

No.	Individuals	JIA Subtype	Gender	Medication History	CyToF	Nanostring
1	**Remission 1018 (T_o)**	Poly RF -	F	etanercept, no MTX	✓	
2	**Remission 1034 (T_o)**	Poly RF -	M	etanercept, no MTX	✓	
3	**Remission 1124 (T_o)**	Poly RF -	F	etanercept, no MTX	✓	
4	**Remission 2022 (T_o)**	Poly RF +	F	etanercept, yes MTX	✓	✓
5	**Remission 2114 (T_o)**	Poly RF -	F	etanercept, no MTX	✓	
6	**Remission 4014 (T_o)**	Poly RF -	F	etanercept, no MTX	✓	
7	**Remission 5121 (T_o)**	Poly RF -	M	etanercept, yes MTX	✓	
8	**Remission 6128 (T_o)**	Poly RF -	M	etanercept, yes MTX	✓	
9	**Remission 7056 (T_o)**	Poly RF -	F	etanercept, no MTX	✓	
10	**Remission 8130 (T_o)**	Poly RF -	F	adalimumab, yes MTX	✓	
11	**Remission 15122 (T_o)**	Poly RF +	F	etanercept, no MTX	✓	
12	**Remission 5127 (T_o)**	Poly RF -	F	etanercept, yes MTX		✓
13	**Remission 4073 (T_o)**	Poly RF +	F	adalimumab, no MTX		✓
1	**Remission 2022 (T_{end})**	Poly RF +	F	etanercept, yes MTX	✓	
2	**Remission 5026 (T_{end})**	Poly RF -	M	etanercept, yes MTX	✓	
3	**Remission 8054 (T_{end})**	Poly RF -	F	etanercept, no MTX	✓	
4	**Remission 8131 (T_{end})**	Poly RF -	M	etanercept, no MTX	✓	
5	**Remission 9086 (T_{end})**	Poly RF -	F	etanercept, no MTX	✓	✓
6	**Remission 9098 (T_{end})**	Poly RF -	F	etanercept, no MTX	✓	
7	**Remission 16111 (T_{end})**	Poly RF +	F	etanercept, yes MTX	✓	
8	**Remission 10081 (T_{end})**	Poly RF +	M	etanercept, yes MTX		✓
9	**Remission 3103 (T_{end})**	Poly RF +	F	adalimumab, no MTX		✓

		Relapse		Remission		Healthy
		T_o (n=12)	T_{end} (n=11)	T_o (n=13)	T_{end} (n=9)	n= 69
**Anti-TNFα Biologics**	**etanercept (%)**	8 (66.7%)	10 (90.9%)	11 (84.6%)	9 (100%)	N.A.
	**adalimumab (%)**	3 (25%)	0 (0%)	2 (15.4%)	0 (0%)	N.A.
	**Infliximab (%)**	1 (8.33%)	1 (9.1%)	0 (0%)	0 (0%)	N.A.
**Methotrexate (concurrent) (%)**		3 (25%)	4 (36.4%)	5 (38.5%)	4 (44.4%)	N.A.
**Rheumatoid Factor+**		0 (0%)	0	3 (23.1%)	4 (44.4%)	N.A.
**Average age (yrs) ± Std Dev**		14.2 ± 4.2	12.5 ± 5.1	9.0 ± 3.4	12.6 ± 4.2	9 ± 3
**Female (n): Male (n)**		11:1	10:1	10:3	6:3	10:59

Supplementary Table S2

Patient ID	Date	Disease	age	Disease Duration (yrs)	Gender	Joints	ESR	CRP	Medication history
RD00444 Pre	25/7/16	Poly JIA, RF+	16.5	11.8	F	5	Not done	Not done	Sulfasalazine, MTX, Folic acid, HCQ
RD00444 Post	10/2/17	Poly JIA, RF+	17.0	12.4	F	0	48	2	MTX, Folic acid, Enbrel
RD05137 Pre	11/3/16	Poly JIA, RF+	10.9	0.1	F	4	26	1.5	-
RD05137 Post	15/9/16	Poly JIA, RF+	11.4	0.6	F	0	7	<0.2	Enbrel, MTX, Folic acid
RD04080 Pre	13/4/16	Oligo JIA, Extended	13.6	2.4	F	4	47	18.2	MTX, Prednisolone, Omeprazole, Folic acid
RD04080 Post	26/10/16	Oligo JIA, Extended	14.1	2.9	F	0	21	5.7	MTX, Folic acid, Enbrel
RD05336 Pre	4/8/16	Oligo JIA, Extended	1.9	0.1	F	8	97	49.3	Brufen
RD05336 Post	10/2/17	Oligo JIA, Extended	2.4	0.6	F	0	8	0.4	Enbrel

Supplementary Table S3

Targets	Metal Channel	Staining	Clone	Antibody Vendor/Catalogue number
Lineage markers				
CD3	139	Surface/Intra	UCHT1	Biolegend (300402)
CD4	148	Surface/Intra	SK3	Biolegend (344625)
CD8	144	Surface	SK1	Biolegend (344727)
CD11b	161	Surface	ICRF44	Biolegend (301302)
CD16	209	Surface	3G8	Fluidigm (3209002B)
CD14	112/114	Surface	TuK4	Thermofisher/Invitrogen (Q10064)
T helper subsets				
IL-4	156	Intra	8D4-8	Biolegend (500707)
IFN-g	168	Intra	B27	Biolegend (506513)
IL-17A	169	Intra	BL168	Biolegend (512302)
IL-21	151	Intra	3A4-N2	Biolegend (513009)
CD161	157	Surface	HP-3G10	Biolegend (339902)
T cell functional				
CD45RA	171	Surface	HI100	Biolegend (304102)
CD69	176	Surface	FN50	Biolegend (310902)
CD28	146	Surface	CD28.2	Biolegend (302923)
CD152 (CTLA4)	155	Intra	BNI3	Biolegend (555851)
CD154 (CD40L)	149	Surface	24-31	Biolegend (310835)
HLA-DR	143	Surface	L243	Biolegend (307612)
LAG3	159	Surface	17B4	Abcam (ab40466)
PD1	147	Surface	EH12.2H7	Biolegend (329941)
Ki67	166	Intra	20Raj1	Thermofisher/ebioscience (14-5699-82)
ICOS	154	Surface	C398.4A	Biolegend (313512)
CD31	172	Surface	WM59	Biolegend (303102)
CD103	142	Surface	B-Ly7	Thermofisher/ebioscience (14-1038-82)
Chemokine receptors				
CXCR3	163	Surface	G025H7	Biolegend (353718)
CXCR5	160	Surface	RF8B2	BD biosciences (552032)
CCR5	145	Surface	NP-6G4	Abcam (ab115738)
CCR6	170	Surface	G034E3	Biolegend (353402)
Treg markers				
CD25	150	Surface	M-A251	BD biosciences (555429)
CD127	153	Surface	A019D5	Biolegend (351302)
FoxP3	165	Intra	PCH10L	Thermofisher/ebioscience (14-4776-82)
GITR	164	Surface	621	Biolegend (311602)
TGF-B (LAP)	175	Surface/Intra	TW4-2F8	Biolegend (349602)
IL-10	158	Intra	JES3-9D7	Biolegend (501402)
Cytokines/Enzymes				
TNFα	152	Intra	Mab11	Biolegend (502902)
IL-6	162	Intra	MQ2-13A5	Thermofisher/ebioscience (16-7069-85)
Granzyme B	173	Intra	CLB-GB11	Abcam (ab103159)
Perforin	174	Intra	B-D48	Abcam (ab47225)
Barcodes				
CD45-A	89	Surface	HI30	Fluidigm (3089003B)
CD45-B,C or D	115, 141, 167	Surface	HI30	Biolegend (304002)
Live/Dead /Singlets				
DNA (Singlets)	191/193	-	Nil	Fluidigm Cell-ID Intercalator-Ir (201192B)
Cisplatin (Live/Dead)	195	-	Nil	Sigma-aldrich (479306-1G)

Supplementary Table S4

Category	Term	Count	%	P-Value	Benjamin
**TCR activation**					
KEGG_PATHWAY	T cell receptor signaling pathway	10	13.2	4.7E-07	3.9E-06
GOTERM_BP_DIRECT	T cell receptor signaling pathway	8	10.5	4.3E-06	3.5E-04
REACTOME_PATHWAY	R-HSA-202424 (Downstream TCR signaling)	6	7.9	5.6E-04	9.4E-03
BIOCARTA	T Cell Receptor Signaling Pathway	5	6.6	4.6E-02	2.4E-01
**Apoptosis**					
UP_KEYWORDS	Apoptosis	15	19.7	7.3E-09	2.6E-07
GOTERM_BP_DIRECT	apoptotic process	14	18.4	1.2E-06	1.4E-04
GOTERM_BP_DIRECT	negative regulation of apoptotic process	11	14.5	3.3E-05	1.8E-03
GOTERM_BP_DIRECT	positive regulation of apoptotic process	10	13.2	7.4E-06	4.6E-04
GOTERM_BP_DIRECT	regulation of apoptotic process	9	11.8	4.8E-06	3.5E-04
KEGG_PATHWAY	Apoptosis	6	7.9	2.9E-04	1.2E-03
GOTERM_BP_DIRECT	apoptotic signaling pathway	5	6.6	2.8E-04	9.7E-03
GOTERM_BP_DIRECT	activation of cysteine-type endopeptidase activity involved in apoptotic process	4	5.3	6.1E-03	9.6E-02
BIOCARTA	Neuropeptides VIP and PACAP inhibit the apoptosis of activated T cells	4	5.3	4.6E-02	2.5E-01
BIOCARTA	Induction of apoptosis through DR3 and DR4/5 Death Receptors	4	5.3	8.2E-02	3.6E-01
**TNF-alpha signalling**					
KEGG_PATHWAY	TNF signaling pathway	15	19.7	6.7E-13	1.0E-10
BIOCARTA	TNFR2 Signaling Pathway	8	10.5	4.8E-07	7.4E-05
BIOCARTA	TNF/Stress Related Signaling	8	10.5	6.2E-06	3.2E-04
GOTERM_BP_DIRECT	tumor necrosis factor-mediated signaling pathway	7	9.2	1.4E-05	8.5E-04
GOTERM_BP_DIRECT	cellular response to tumor necrosis factor	5	6.6	1.5E-03	3.6E-02
**NF-kB signalling**					
KEGG_PATHWAY	Toll-like receptor signaling pathway	11	14.5	4.8E-08	5.3E-07
KEGG_PATHWAY	NF-kappa B signaling pathway	11	14.5	7.0E-09	1.2E-07
KEGG_PATHWAY	NOD-like receptor signaling pathway	10	13.2	1.7E-09	5.2E-08
GOTERM_BP_DIRECT	positive regulation of NF-kappaB transcription factor activity	10	13.2	7.5E-09	2.0E-06
GOTERM_BP_DIRECT	positive regulation of I-kappaB kinase/NF-kappaB signaling	9	11.8	5.9E-07	7.6E-05
KEGG_PATHWAY	RIG-I-like receptor signaling pathway	9	11.8	2.7E-07	2.7E-06
REACTOME_PATHWAY	R-HSA-445989 (TAK1 activates NFkB by phosphorylation and activation of IKKs complex)	8	10.5	7.8E-10	2.0E-07
REACTOME_PATHWAY	R-HSA-168638 (NOD1/2 Signaling Pathway)	7	9.2	1.2E-07	1.5E-05
KEGG_PATHWAY	Cytosolic DNA-sensing pathway	7	9.2	2.9E-05	1.6E-04
BIOCARTA	NF-kB Signaling Pathway	7	9.2	4.8E-05	1.5E-03
REACTOME_PATHWAY	R-HSA-1810476 (RIP-mediated NFkB activation via ZBP1)	6	7.9	2.2E-07	1.8E-05
REACTOME_PATHWAY	R-HSA-933542 (TRAF6 mediated NF-kB activation)	6	7.9	4.5E-07	2.8E-05
REACTOME_PATHWAY	R-HSA-2871837 (FCERI mediated NF-kB activation)	6	7.9	2.2E-03	2.7E-02
GOTERM_BP_DIRECT	nucleotide-binding oligomerization domain containing signaling pathway	5	6.6	4.3E-06	3.3E-04
GOTERM_BP_DIRECT	I-kappaB kinase/NF-kappaB signaling	5	6.6	1.5E-04	6.4E-03
GOTERM_BP_DIRECT	NIK/NF-kappaB signaling	4	5.3	3.2E-03	6.1E-02
GOTERM_BP_DIRECT	negative regulation of NF-kappaB transcription factor activity	4	5.3	3.9E-03	7.2E-02
REACTOME_PATHWAY	R-HSA-5668541 (TNFR2 non-canonical NF-kB pathway)	4	5.3	1.1E-02	8.9E-02
**MAPK signalling**					
KEGG_PATHWAY	MAPK signaling pathway	9	11.8	2.8E-03	8.9E-03
BIOCARTA	MAPKinase Signaling Pathway	7	9.2	5.1E-02	2.6E-01
GOTERM_BP_DIRECT	MAPK cascade	5	6.6	3.0E-02	3E-01
GOTERM_BP_DIRECT	positive regulation of MAPK cascade	4	5.3	5.7E-03	9.1E-02
GOTERM_BP_DIRECT	activation of MAPK activity	4	5.3	1.2E-02	1.6E-01

Supplementary Table S5

Category	Term	Count	%	P-Value	Benjamin
**TCR activation**					
KEGG_PATHWAY	T cell receptor signaling pathway	12	13.3	1.8E-08	1.6E-07
GOTERM_BP_DIRECT	T cell receptor signaling pathway	10	11.1	8.8E-08	1.4E-05
REACTOME_PATHWAY	R-HSA-202424 (Downstream TCR signaling)	7	7.8	1.4E-04	3.4E-03
BIOCARTA	T Cell Receptor Signaling Pathway	7	7.8	4.6E-03	5.2E-02
**Apoptosis**					
UP_KEYWORDS	Apoptosis	18	20.0	1.2E-10	6.1E-09
GOTERM_BP_DIRECT	apoptotic process	17	18.9	4.2E-08	7.2E-06
GOTERM_BP_DIRECT	regulation of apoptotic process	12	13.3	1.6E-08	3.2E-06
GOTERM_BP_DIRECT	negative regulation of apoptotic process	12	13.3	2.8E-05	1.3E-03
GOTERM_BP_DIRECT	positive regulation of apoptotic process	11	12.2	4.2E-06	3.2E-04
KEGG_PATHWAY	Apoptosis	6	6.7	6.7E-04	2.5E-03
GOTERM_BP_DIRECT	apoptotic signaling pathway	5	5.6	5.5E-04	1.3E-02
GOTERM_BP_DIRECT	activation of cysteine-type endopeptidase activity involved in apoptotic process	5	5.6	9.8E-04	2.1E-02
GOTERM_BP_DIRECT	positive regulation of apoptotic signaling pathway	4	4.4	3.0E-04	9.3E-03
GOTERM_BP_DIRECT	extrinsic apoptotic signaling pathway via death domain receptors	4	4.4	1.1E-03	2.3E-02
GOTERM_BP_DIRECT	intrinsic apoptotic signaling pathway in response to DNA damage	4	4.4	2.0E-03	3.7E-02
BIOCARTA	Neuropeptides VIP and PACAP inhibit the apoptosis of activated T cells	4	4.4	6.8E-02	3.1E-01
**TNF-alpha signalling**					
KEGG_PATHWAY	TNF signaling pathway	16	17.8	5.3E-13	2.7E-11
BIOCARTA	TNFR2 Signaling Pathway	9	10.0	7.1E-08	1.1E-05
BIOCARTA	TNF/Stress Related Signaling	8	8.9	1.9E-05	7.6E-04
GOTERM_BP_DIRECT	tumor necrosis factor-mediated signaling pathway	7	7.8	3.8E-05	1.7E-03
GOTERM_BP_DIRECT	cellular response to tumor necrosis factor	6	6.7	3.0E-04	9.3E-03
GOTERM_BP_DIRECT	regulation of tumor necrosis factor-mediated signaling pathway	4	4.4	5.3E-04	1.3E-02
REACTOME_PATHWAY	R-HSA-5357905 (Regulation of TNFR1 signaling)	4	4.4	2.2E-03	2.7E-02
**NF-kB signalling**					
KEGG_PATHWAY	Toll-like receptor signaling pathway	14	15.6	1.4E-10	3.6E-09
GOTERM_BP_DIRECT	positive regulation of I-kappaB kinase/NF-kappaB signaling	12	13.3	8.6E-10	2.4E-07
KEGG_PATHWAY	NF-kappa B signaling pathway	12	13.3	3.0E-09	3.8E-08
KEGG_PATHWAY	NOD-like receptor signaling pathway	11	12.2	4.1E-10	7.9E-09
GOTERM_BP_DIRECT	positive regulation of NF-kappaB transcription factor activity	11	12.2	2.1E-09	4.8E-07
KEGG_PATHWAY	RIG-I-like receptor signaling pathway	11	12.2	4.9E-09	5.7E-08
KEGG_PATHWAY	Cytosolic DNA-sensing pathway	10	11.1	3.5E-08	3.0E-07
REACTOME_PATHWAY	R-HSA-445989 (TAK1 activates NFkB by phosphorylation and activation of IKKs complex)	8	8.9	2.6E-09	7.5E-07
REACTOME_PATHWAY	R-HSA-168638 (NOD1/2 Signaling Pathway)	8	8.9	1.0E-08	1.5E-06
BIOCARTA	NF-kB Signaling Pathway	7	7.8	1.2E-04	3.9E-03
REACTOME_PATHWAY	R-HSA-1810476 (RIP-mediated NFkB activation via ZBP1)	6	6.7	5.0E-07	4.8E-05
REACTOME_PATHWAY	R-HSA-933542 (TRAF6 mediated NF-kB activation)	6	6.7	1.0E-06	7.5E-05
REACTOME_PATHWAY	R-HSA-2871837 (FCERI mediated NF-kB activation)	6	6.7	4.5E-03	4.9E-02
GOTERM_BP_DIRECT	nucleotide-binding oligomerization domain containing signaling pathway	5	5.6	8.6E-06	5.4E-04
GOTERM_BP_DIRECT	TRIF-dependent toll-like receptor signaling pathway	5	5.6	1.4E-05	7.9E-04
GOTERM_BP_DIRECT	I-kappaB kinase/NF-kappaB signaling	5	5.6	2.9E-04	9.2E-03
GOTERM_BP_DIRECT	NIK/NF-kappaB signaling	5	5.6	4.1E-04	1.1E-02
REACTOME_PATHWAY	R-HSA-5357956 (TNFR1-induced NFkappaB signaling pathway)	4	4.4	1.7E-03	2.6E-02
GOTERM_BP_DIRECT	negative regulation of NF-kappaB transcription factor activity	4	4.4	6.3E-03	9.2E-02
REACTOME_PATHWAY	R-HSA-5668541 (TNFR2 non-canonical NF-kB pathway)	4	4.4	1.7E-02	1.3E-01
**MAPK signalling**					
KEGG_PATHWAY	MAPK signaling pathway	9	10.0	8.4E-03	2.3E-02
GOTERM_BP_DIRECT	MAPK cascade	7	7.8	2.7E-03	4.6E-02
BIOCARTA	MAPKinase Signaling Pathway	7	7.8	9.7E-02	3.8E-01
GOTERM_BP_DIRECT	activation of MAPK activity	5	5.6	2.5E-03	4.4E-02
GOTERM_BP_DIRECT	positive regulation of MAPK cascade	4	4.4	9.1E-03	1.2E-01
REACTOME_PATHWAY	R-HSA-5673001 (RAF/MAP kinase cascade)	4	4.4	6.4E-02	3.4E-01

Supplementary Table S6

Category	Term	Count	%	P-Value	Benjamini
**TCR Activation**					
KEGG_PATHWAY	T cell receptor signaling pathway	8	15.1	3.2E-06	2.8E-05
GOTERM_BP_DIRECT	T cell receptor signaling pathway	7	13.2	6.2E-06	5.8E-04
REACTOME_PATHWAY	R-HSA-202424 (Downstream TCR signaling)	5	9.4	1.2E-03	2.2E-02
BIOCARTA	T Cell Receptor Signaling Pathway	4	7.5	4.9E-02	2.1E-01
**Apoptosis**					
GOTERM_BP_DIRECT	apoptotic process	14	26.4	1.2E-08	3.8E-06
UP_KEYWORDS	Apoptosis	13	24.5	6.9E-09	2.9E-07
GOTERM_BP_DIRECT	regulation of apoptotic process	9	17.0	2.7E-07	4.3E-05
GOTERM_BP_DIRECT	positive regulation of apoptotic process	7	13.2	3.2E-04	1.2E-02
GOTERM_BP_DIRECT	negative regulation of apoptotic process	7	13.2	2.7E-03	6.8E-02
KEGG_PATHWAY	Apoptosis	6	11.3	4.1E-05	2.7E-04
GOTERM_BP_DIRECT	activation of cysteine-type endopeptidase activity involved in apoptotic process	5	9.4	1.3E-04	5.6E-03
BIOCARTA	Neuropeptides VIP and PACAP inhibit the apoptosis of activated T cells	4	7.5	1.2E-02	7.5E-02
BIOCARTA	Induction of apoptosis through DR3 and DR4/5 Death Receptors	4	7.5	2.3E-02	1.2E-01
**TNF-alpha Signalling**					
KEGG_PATHWAY	TNF signaling pathway	11	20.8	7.5E-10	2.2E-08
BIOCARTA	TNF/Stress Related Signaling	8	15.1	1.7E-07	1.0E-05
BIOCARTA	TNFR2 Signaling Pathway	6	11.3	1.3E-05	4.0E-04
GOTERM_BP_DIRECT	cellular response to tumor necrosis factor	4	7.5	4.8E-03	9.9E-02
GOTERM_BP_DIRECT	tumor necrosis factor-mediated signaling pathway	4	7.5	5.8E-03	1.1E-01
**NF-KB Signalling**					
KEGG_PATHWAY	NF-kappa B signaling pathway	11	20.8	1.0E-10	1.5E-08
KEGG_PATHWAY	Toll-like receptor signaling pathway	11	20.8	7.5E-10	2.2E-08
KEGG_PATHWAY	RIG-I-like receptor signaling pathway	10	18.9	3.5E-10	1.7E-08
GOTERM_BP_DIRECT	positive regulation of I-kappaB kinase/NF-kappaB signaling	10	18.9	1.4E-09	1.3E-06
GOTERM_BP_DIRECT	positive regulation of NF-kappaB transcription factor activity	8	15.1	1.7E-07	3.1E-05
KEGG_PATHWAY	NOD-like receptor signaling pathway	9	17.0	1.4E-09	3.3E-08
REACTOME_PATHWAY	R-HSA-168638 (NOD1/2 Signaling Pathway)	7	13.2	1.2E-08	2.7E-06
REACTOME_PATHWAY	R-HSA-933542 (TRAF6 mediated NF-kB activation)	6	11.3	6.6E-08	7.5E-06
REACTOME_PATHWAY	R-HSA-445989 (TAK1 activates NFkB by phosphorylation and activation of IKKs complex)	6	11.3	2.2E-07	1.6E-05
BIOCARTA	NF-kB Signaling Pathway	6	11.3	4.8E-05	1.2E-03
REACTOME_PATHWAY	R-HSA-1810476 (RIP-mediated NFkB activation via ZBP1)	5	9.4	2.2E-06	1.0E-04
REACTOME_PATHWAY	R-HSA-2871837 (FCERI mediated NF-kB activation)	5	9.4	3.6E-03	4.7E-02
GOTERM_BP_DIRECT	nucleotide-binding oligomerization domain containing signaling pathway	4	7.5	6.1E-05	3.6E-03
GOTERM_BP_DIRECT	toll-like receptor signaling pathway	4	7.5	7.8E-05	4.0E-03
GOTERM_BP_DIRECT	TRIF-dependent toll-like receptor signaling pathway	4	7.5	8.7E-05	4.3E-03
GOTERM_BP_DIRECT	NIK/NF-kappaB signaling	4	7.5	1.1E-03	3.4E-02
**MAPK Signalling**					
KEGG_PATHWAY	MAPK signaling pathway	8	15.1	1.0E-03	4.6E-03
BIOCARTA	MAPKinase Signaling Pathway	7	13.2	4.9E-03	3.9E-02
BIOCARTA	Human Cytomegalovirus and Map Kinase Pathways	4	7.5	3.6E-03	3.3E-02
GOTERM_BP_DIRECT	activation of MAPK activity	4	7.5	4.4E-03	9.4E-02
GOTERM_BP_DIRECT	MAPK cascade	4	7.5	4.7E-02	4.3E-01
