# Supplementary material for: Immunome perturbation is present in patients with juvenile idiopathic arthritis who are in remission and will relapse upon anti-TNFα withdrawal
Source: Ann Rheum Dis. 2019 Sep 20;78(12):1712–21. doi: 10.1136/annrheumdis-2019-216059 (PMC6900250; doi:10.1136/annrheumdis-2019-216059)

Supplementary Figure S1

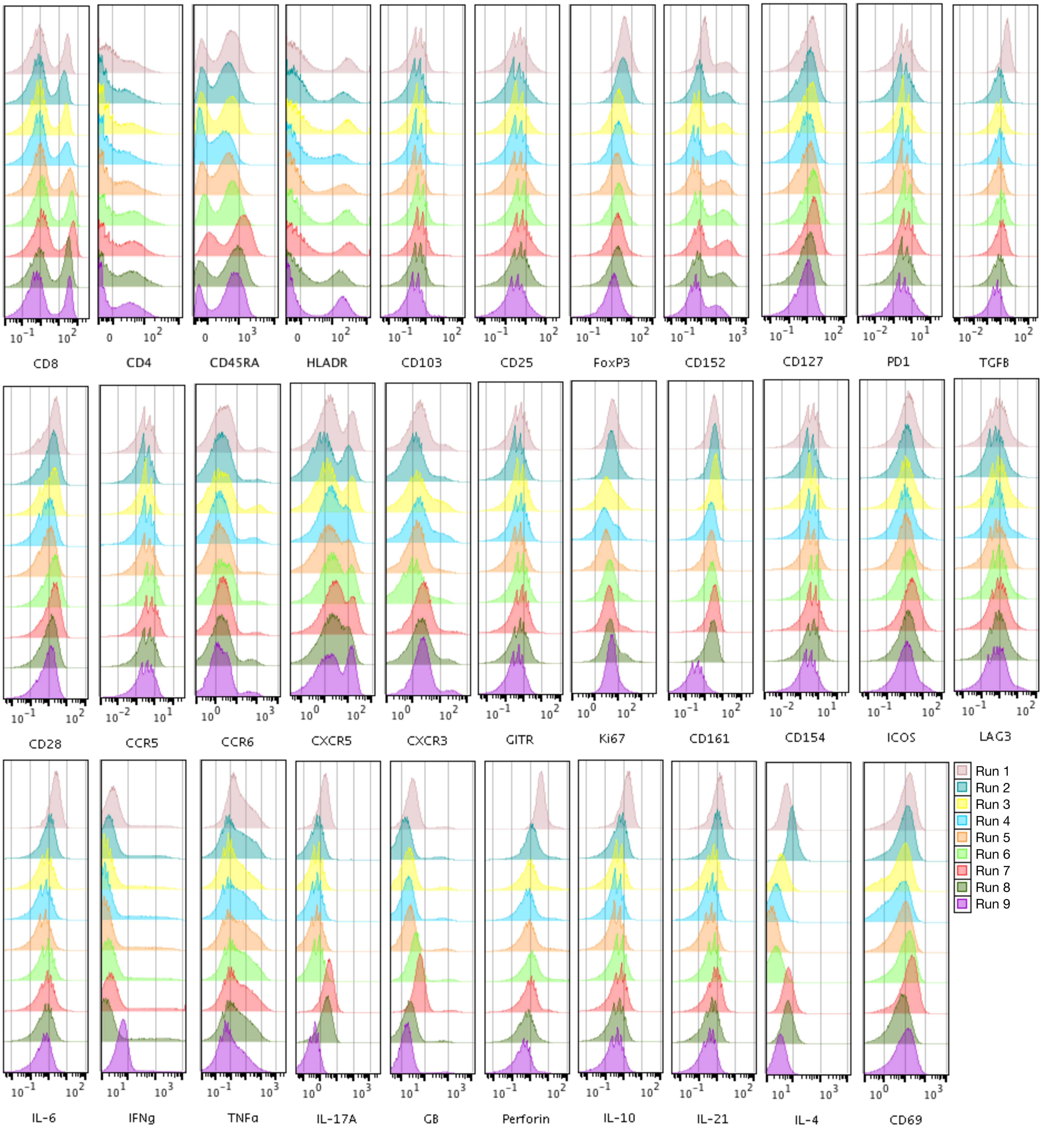

Supplementary Figure S2

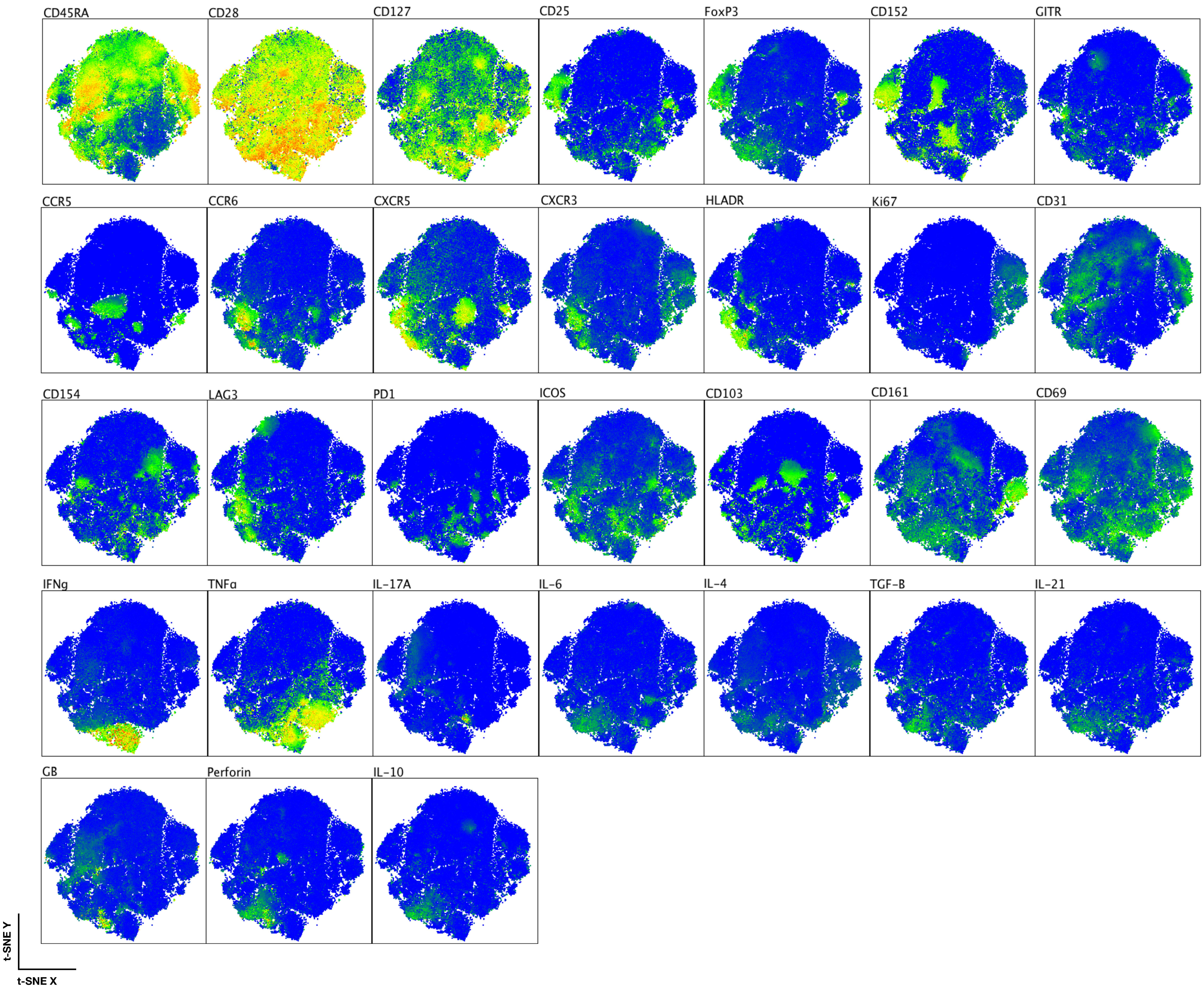

Supplementary Figure S3

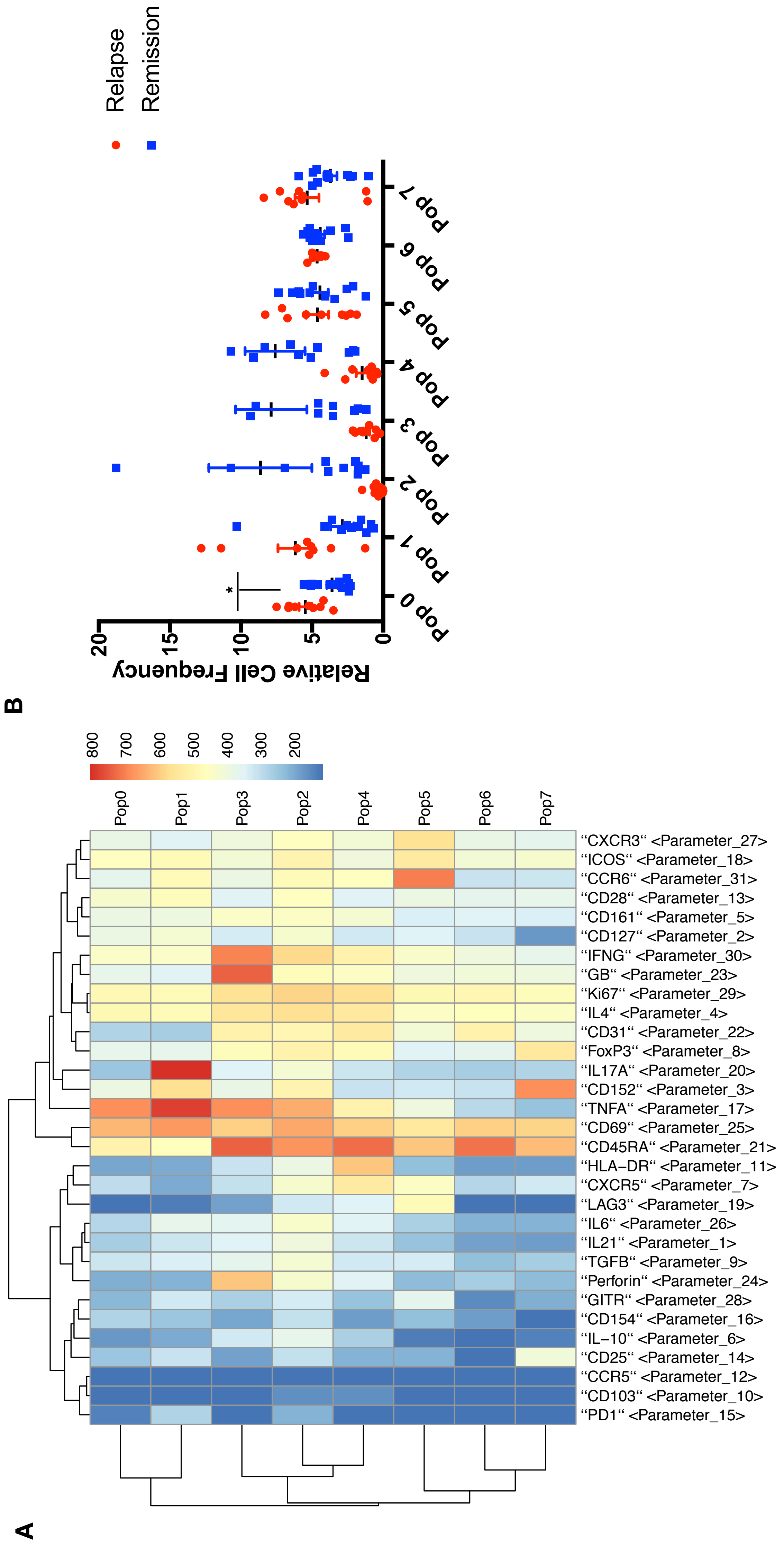

Supplementary Figure S4

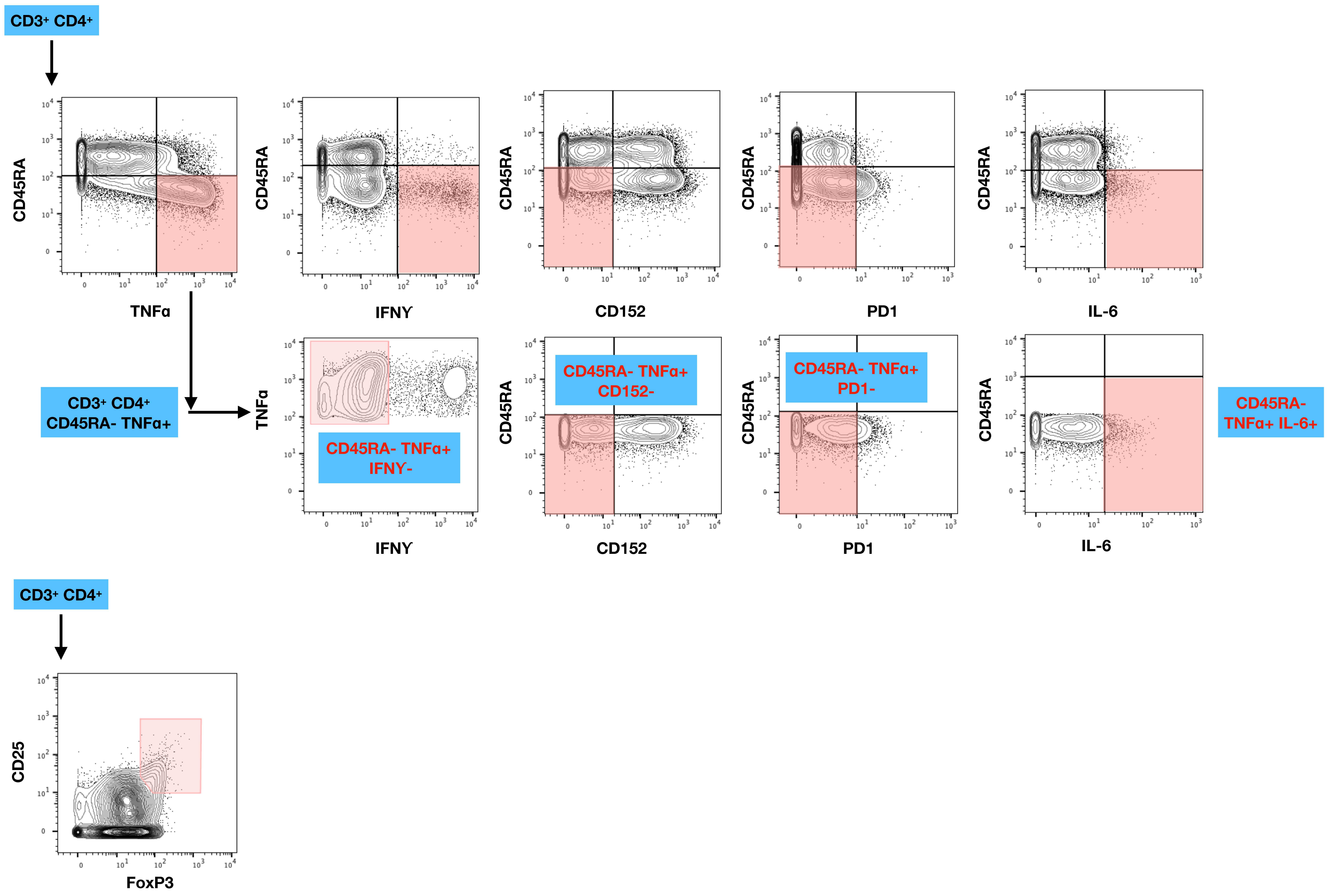

Supplementary Figure S5

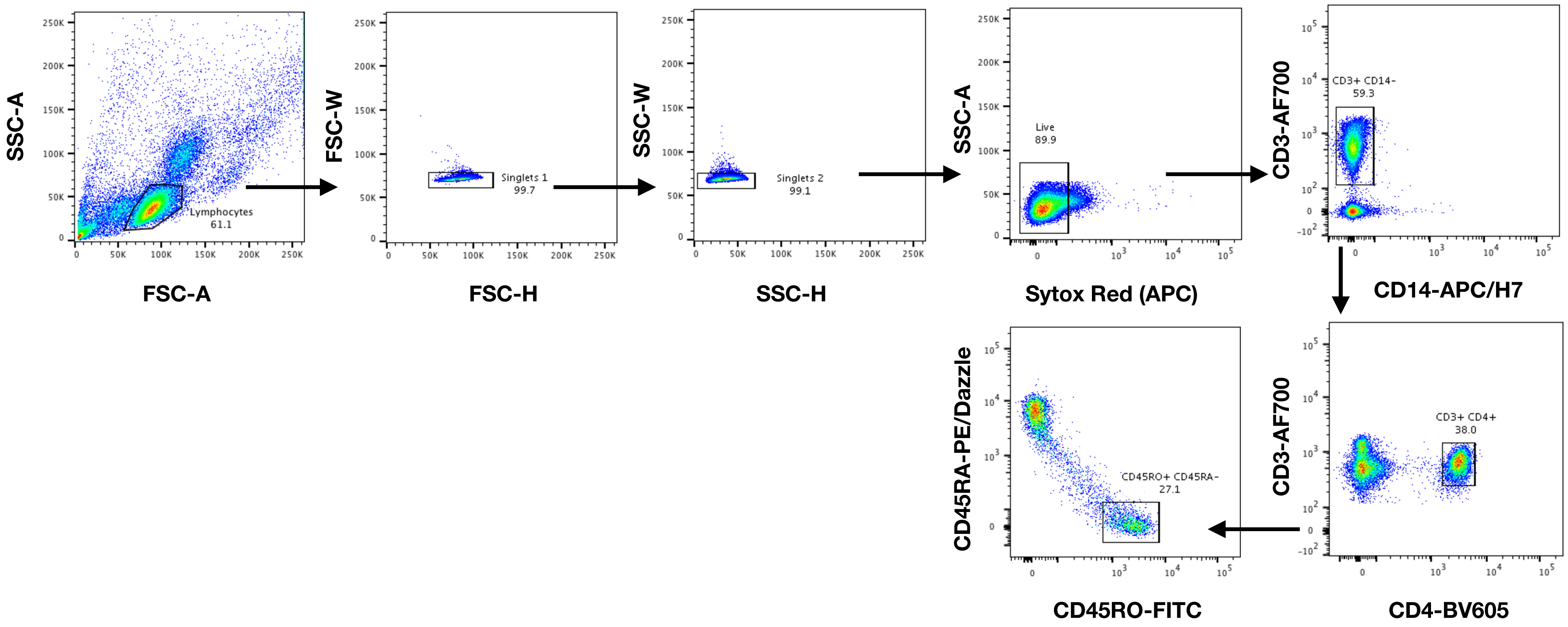

Supplementary Figure S6

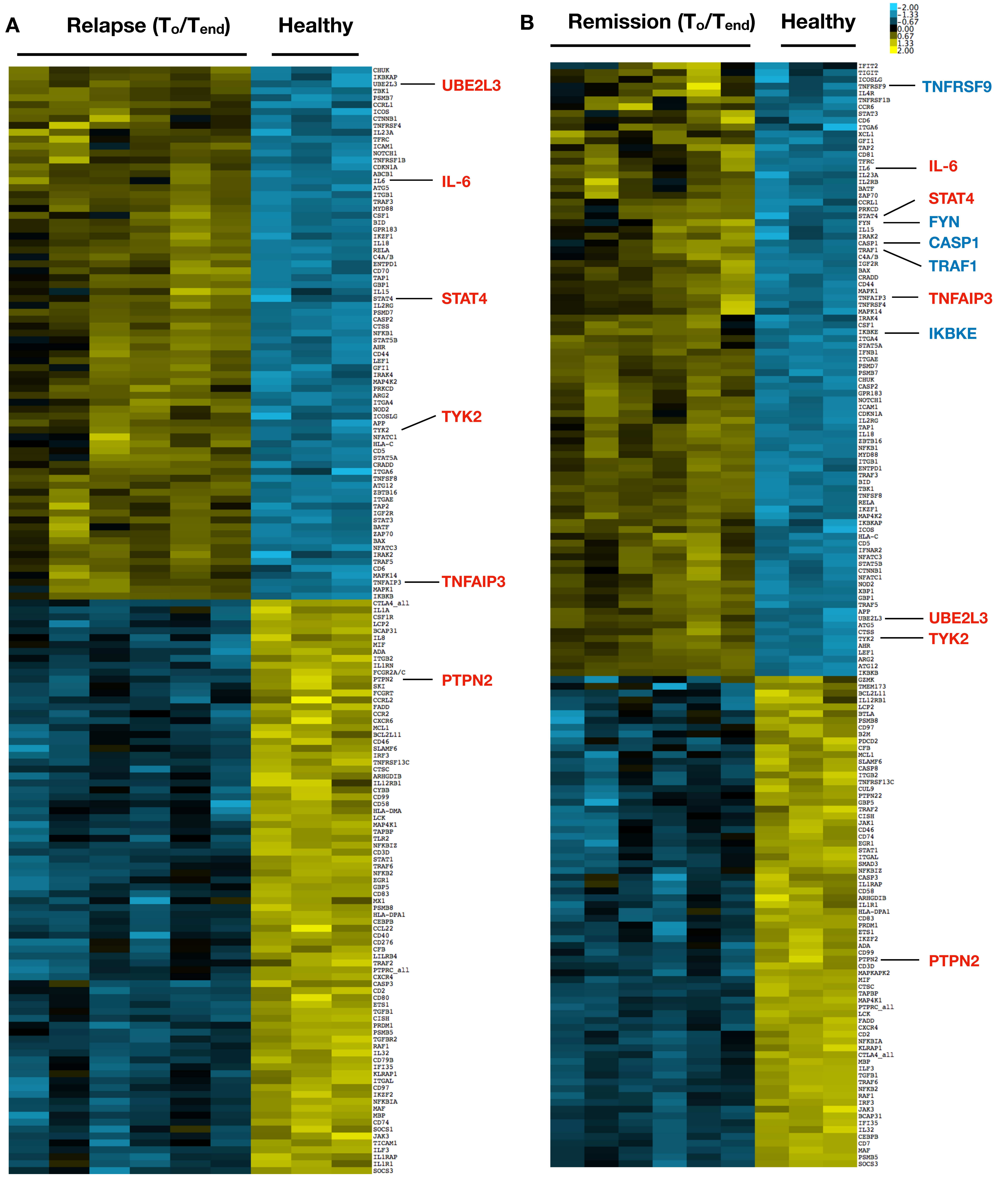

Supplementary Figure S7

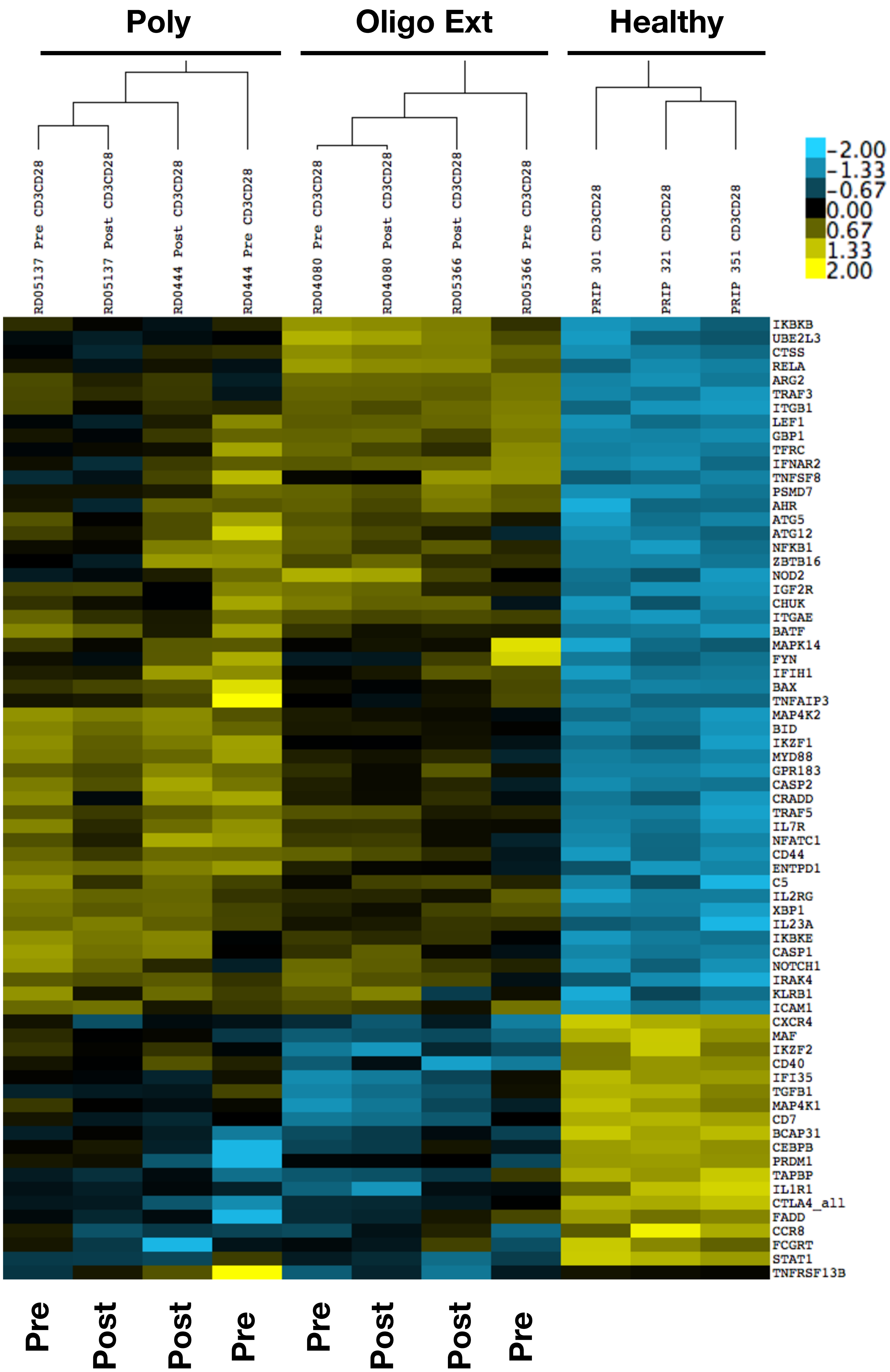

Supplementary Figure S8

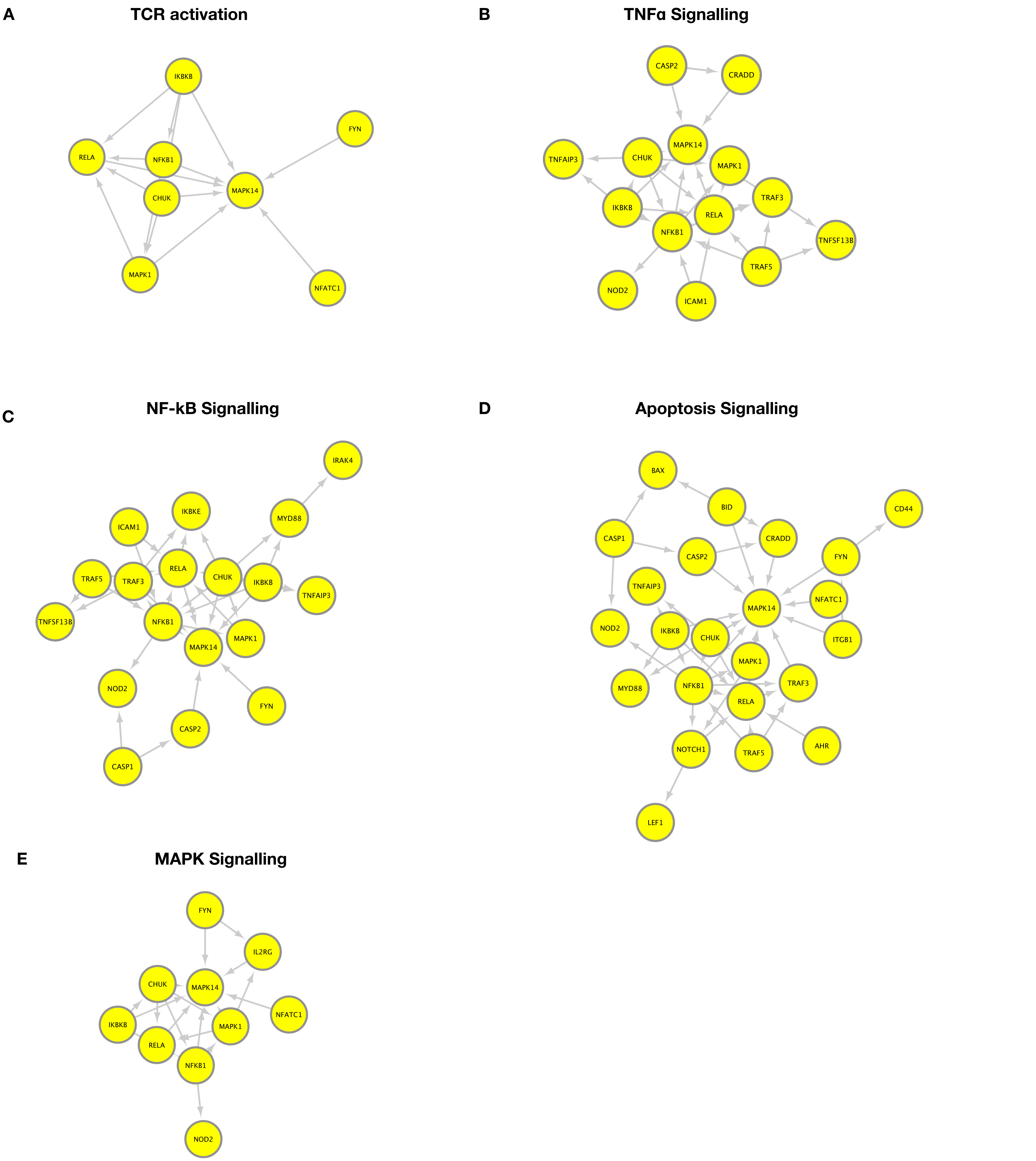

Supplementary Figure S9

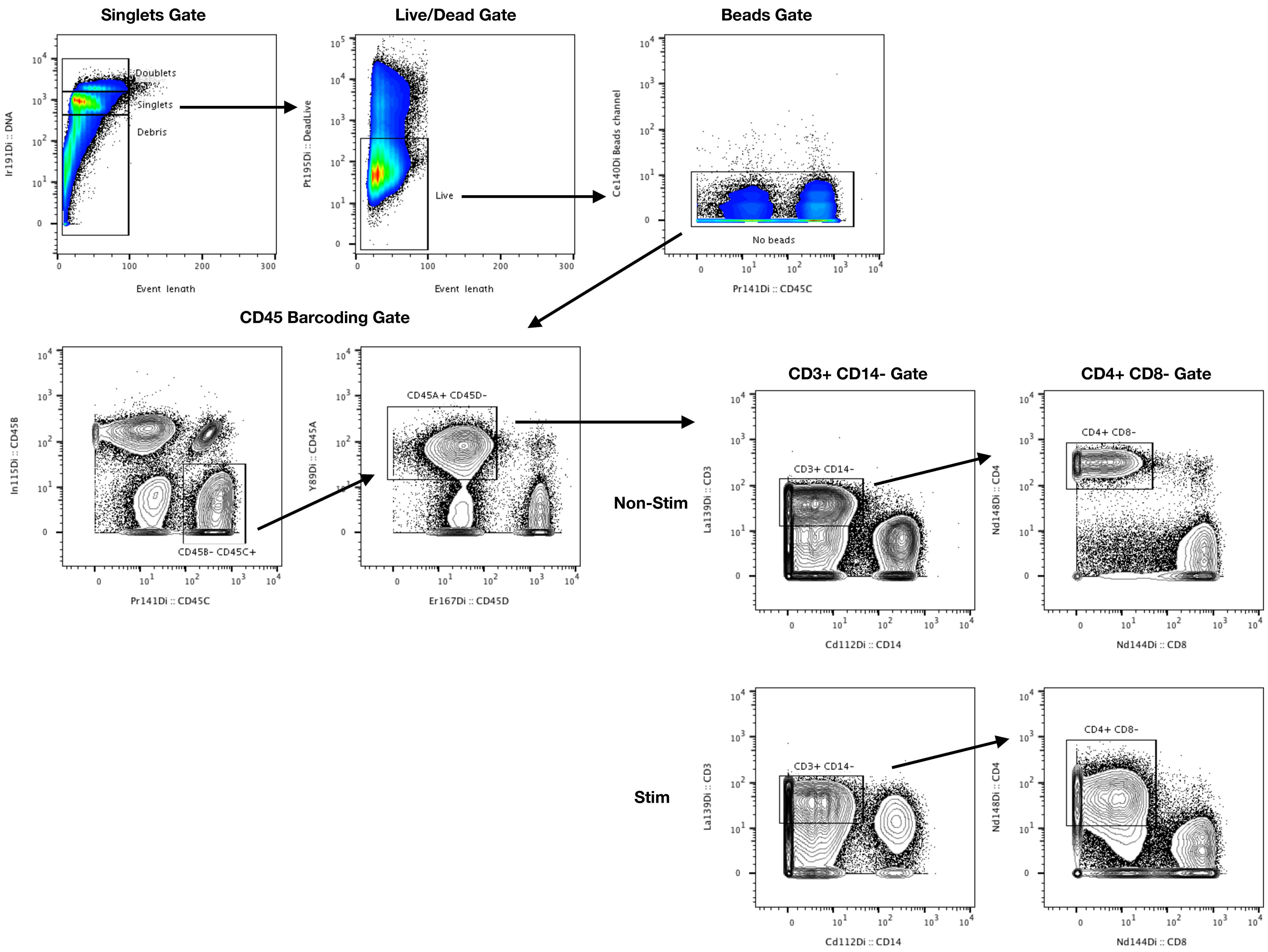

Supplement: Supplementary data [file annrheumdis-2019-216059supp001.pdf]
